# Pressure-Dependent Clustering in Ionic-Liquid-Poly (Vinylidene Fluoride) Mixtures: An Infrared Spectroscopic Study

**DOI:** 10.3390/nano11082099

**Published:** 2021-08-18

**Authors:** Teng-Hui Wang, Wei-Xiang Wang, Hai-Chou Chang

**Affiliations:** Department of Chemistry, National Dong Hwa University, Shoufeng, Hualien 974, Taiwan; 810712101@gms.ndhu.edu.tw (T.-H.W.); 610812112@gms.ndhu.edu.tw (W.-X.W.)

**Keywords:** PVdF, [HEMIm][TFSI], choline [TFSI], high pressures

## Abstract

The nanostructures of ionic liquids (ILs) have been the focus of considerable research attention in recent years. Nevertheless, the nanoscale structures of ILs in the presence of polymers have not been described in detail at present. In this study, nanostructures of ILs disturbed by poly(vinylidene fluoride) (PVdF) were investigated via high-pressure infrared spectra. For 1-(2-hydroxyethyl)-3-methylimidazolium bis(trifluoromethanesulfonyl)imide ([HEMIm][TFSI])-PVdF mixtures, non-monotonic frequency shifts of the C^4,5^-H vibrations upon dilution were observed under ambient pressure. The experimental results suggest the presence of microheterogeneity in the [HEMIm][TFSI] systems. Upon compression, PVdF further influenced the local structure of C^4,5^–H via pressure-enhanced IL–PVdF interactions; however, the local structures of C^2^–H and hydrogen-bonded O–H were not affected by PVdF under high pressures. For choline [TFSI]–PVdF mixtures, PVdF may disturb the local structures of hydrogen-bonded O–H. In the absence of the C^4,5^–H⋯anion and C^2^–H⋯anion in choline [TFSI]–PVdF mixtures, the O–H group becomes a favorable moiety for pressure-enhanced IL–PVdF interactions. Our results indicate the potential of high-pressure application for designing pressure-dependent electronic switches based on the possible changes in the microheterogeneity and electrical conductivity in IL-PVdF systems under various pressures.

## 1. Introduction

As a semi-crystalline polymer, polyvinylidene fluoride (PVdF) consists of five main phase structures, namely α, β, γ, δ, and ε phases [1,2,3,4,5,6]. Owing to the specific conformation of the carbon chain backbone, considerable attention has been paid to the β structure of PVdF, which is known to have piezo-, pyro-, and ferroelectric applications [1,2,3,4,5,6,7,8,9]. Unlike piezoelectric ceramics, piezoelectric polymers such as PVdF are flexible, which may be crucial in the development of wearable electronic mobile devices in the future [1,2,3,4,5,6]. PVdF and its derivative copolymer (as well as piezoelectric ceramic–PVdF composites) may be promising candidates for tactile sensors, pressure sensors, shock sensors, and thermal measurement devices [1,2,3,4,5,6,7,8]. Following polymerization from 1,1-difluoroethylene (CH_2_=CF_2_), PVdF with a >CF_2_ moiety may interact with cations of ionic liquids (ILs) [10,11,12]. PVdF does not easily dissolve in many traditional non-toxic solvents, partly because of its outstanding chemical resistance; the addition of a plasticizer may further enhance the flexibility of PVdF-related complexes. The incorporation of ILs into PVdF may modify its physical properties [11]. IL-PVdF mixtures exhibit excellent electrical conductivity, high transparency, and increased ductility. Good piezoelectric properties, surface roughness values, and degrees of crystallinity are also observed for IL-PVdF mixtures [10,11]. The addition of ILs can help solve the problem of static charge accumulation on the surfaces of pure polymers.

Ionic compounds with melting temperatures below the boiling point of water (ILs) are widely used in industry because of their non-volatile, dispersive, and ionic-conductive properties [13,14,15,16,17,18]. A critical analysis of the structures and properties of ionic liquids was performed by Cabrita’s group [13] recently. The interactions between cations and anions in ILs include forces such as electrostatic attraction, van der Waals association, and hydrogen-bonding interactions [13,14,15,16,17,19,20,21,22,23]. The interplay of various interactions in ILs may determine the formation of nanoscale supramolecular domains in bulk ILs. Indeed, the effects of cation–anion associations, such as on viscosity, may influence the properties of ILs. Recently, the mechanisms and properties of ILs that allow for their use as lubricants were extensively reviewed by Calandra et al. [24]. ILs can also be suitable plasticizers to form polymer–salt complexes from PVdF [10,11]. As reported in the literature, the >CF_2_ moiety in PVdF may interact with the IL cation. For example, >CF_2_ groups in the PVdF chain can interact with the imidazolium C–H of ILs [10,11]. Nevertheless, studies on imidazolium equipped with hydroxyl groups are scarce. Ludwig’s group [25] found that ILs with cations containing –C_2_H_4_OH group ILs tend to form cationic clusters. Investigations of ILs with cations containing hydroxyl moieties may shed light on cation–anion clusters (caused by electrostatic forces and weak hydrogen bond interactions) and cation–cation associations (attributed to the influence of traditional hydrogen bonds). This is because the ions in ILs are composed of clusters of various sizes owing to various interactions.

Vibrational spectra, such as mid-infrared spectra, may provide information on the environmental changes of functional groups upon blending. Infrared (IR) spectra were used to characterize ion-modified materials in the past. For example, Ivanov et al. [26,27] used a nondestructive infrared technique to inspect ion-modified polymeric materials. Hydroxyl groups are IR-sensitive to changes in local environments, and the formation of O–H hydrogen bonds can be easily detected by IR techniques. Moreover, C–H can act as a proton donor to form a weak hydrogen bond. When associated with proton acceptor X to form weak hydrogen bonds (C–H⋯X), C–H covalent bonds sometimes shorten themselves through a blueshift in frequency [20,28,29,30,31]. In this work, we investigated the pressure-dependent local structures of ILs with both O–H and imidazolium C–H, which are IR-detectable.

Combining the IR technique and high pressures may result in pressure-enhanced interactions. As reported in previous studies [32,33,34,35,36], the associated structures of ILs were influenced by the addition of PEO [32] and DNA [33] under high pressure. As the pressure was applied, the polymer (PEO or DNA) disturbed the clustering of the ILs, as polymers and ILs interact with specific pressure-enhanced forces. In the current study, two types of ILs (TFSI anion and two types of cations containing hydroxyethyl groups) were used. Upon blending with PVdF, the conformation and clustering change were studied using the IR technique at various pressures.

## 2. Materials and Methods

IL-PVdF mixtures were prepared using poly(vinylidene fluoride) (PVdF, average MW ~ 534,000, Sigma-Aldrich, St. Louis, MO, USA), choline bis(trifluoromethylsulfonyl)imide (choline [TFSI], 99%, IOLITEC, Heilbronn, Germany), 1-(2-hydroxyethyl)-3-methylimidazolium bis(trifluoromethylsulfonyl)imide ([HEMIm][TFSI], 99%, UniRegion Bio-Tech, Taoyuan, Taiwan), and N, N-dimethylformamide (DMF, ≥99.9%, Sigma-Aldrich, St. Louis, MO, USA). The structures of [HEMIm][TFSI], choline [TFSI], and PVdF are shown in Figure S1 (Supplementary Materials). The numbering of imidazolium atoms is shown in Figure S1. Mixtures of IL–PVdF containing 10, 20, 30, 40, and 50 wt% of IL were prepared with various weight percentages of IL and PVdF and suitable amounts of DMF were added as the solvent. The solutions were sonicated at room temperature (25 °C) and then stirred at 50 °C under vacuum. The solvent (DMF) was removed under vacuum and the samples were kept under light in air for at least one day to remove the residual solvent. The samples were further dried at 155 °C using a moisture analyzer (MS-70, A&D Company, Tokyo, Japan) before spectral measurements were performed. The removal of DMF was confirmed by checking the disappearance of the DMF absorption in the IR spectra.

High pressures (up to ~2 GPa) were generated using a diamond anvil cell (DAC) equipped with two type IIa diamonds with a culet size of 0.6 mm. In the laboratory, we utilized a Fourier-transform (FT) spectrophotometer (Spectrum RXI, Perkin-Elmer, Naperville, IL, USA) combined with a beam condenser to obtain the IR spectra. The beam condenser was combined with a spectrometer to enhance the intensity of the IR beam. The absorption spectra of the samples were measured and subtracted from those of the DAC to eliminate absorption by the diamond anvils. A 0.25-mm-thick Inconel gasket with a 0.3 mm diameter hole was prepared as the sample holder. Transparent CaF_2_ crystals were placed in the sample holder before the samples were inserted to avoid the saturation of the IR bands. The pressure calibration followed Wong’s method [37,38].

## 3. Results and Discussion

**Figure 1:**
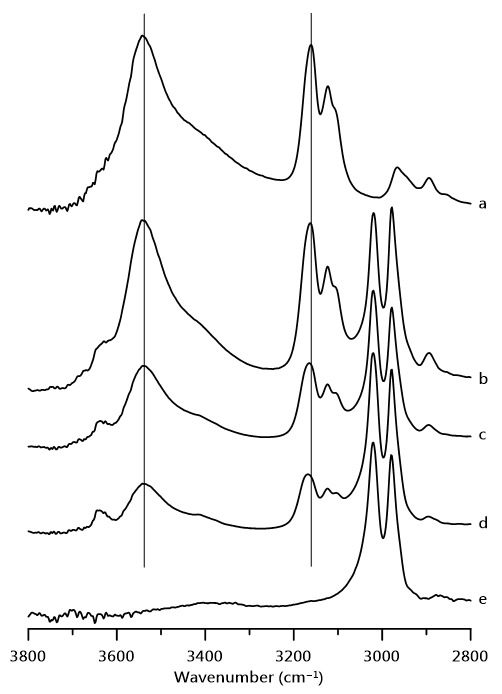
IR spectra of (a) pure [HEMIm][TFSI] and mixture of PVdF containing (b) 30, (c) 20, and (d) 10 wt% [HEMIm][TFSI] and (e) pure PVdF at ambient pressure.

Figure 1 shows infrared spectra of (Figure 1a) pure [HEMIm][TFSI] mixtures of [HEMIm][TFSI]-PVdF containing (Figure 1b) 30, (Figure 1c) 20, and (Figure 1d) 10 wt% [HEMIm][TFSI], as well as (Figure 1e) pure PVdF obtained at ambient pressure. The IR spectra of pure [HEMIm][TFSI] in Figure 1a show broad O–H absorptions, two main imidazolium C–H bands, and several alkyl C–H peaks at 3300–3700, 3050–3250, and 2800–3000 cm^−1^, respectively, at ambient pressure [25,32,33,34,35,36]. As shown in Figure 1a, the O–H absorption also displays a shoulder peak at ca. 3430 cm^−1^. The two imidazolium C–H bands at 3162 and 3123 cm^−1^ represent the vibrational absorptions of the C^4,5^–H and C^2^–H groups, respectively, whereas the C^2^–H peak of pure [HEMIm][TFSI] reveals one shoulder band at 3109 cm^−1^ in Figure 1a. The pure PVdF vibrational spectra in Figure 1e show two main C–H absorptions, which are attributed to asymmetric and symmetric PVdF C–H stretching vibrations at 3021 and 2985 cm^−1^, respectively [6,7,8]. Based on the work by Ludwig’s group [25], O–H stretching absorptions of pure [HEMIm][TFSI] at ca. 3540 and 3430 cm^−1^ are related to the O–H⋯O=S and O–H⋯O–H hydrogen bond interactions. As indicated in Figure 1a, the spectral features of pure [HEMIm][TFSI] in the region of 3300–3700 cm^−1^ (O–H bands) suggest that the pure [HEMIm][TFSI] contains a cation–anion hydrogen bonding association (3539 cm^−1^) and cationic clusters (3430 cm^−1^) at atmospheric pressure. Minor absorptions in the 3600–3800 cm^−1^ region become obvious in [HEMIm][TFSI]-PVdF mixtures containing 30, 20, and 10 wt% of [HEMIm][TFSI], as shown in Figure 1b–d, respectively. The minor O–H peak—for example at 3634 cm^−1^ for the 30 wt% [HEMIm][TFSI]-PVdF mixture in Figure 1b—may be assigned to the partially free O–H moiety of the cation. In the literature, the formation of H-bonding causes a redshift (lower shift) in the frequency of O–H stretching vibrations [20,23,28,29]. As such, the breakage of H-bonding may induce a blueshift (higher shift) in frequency for the O–H vibrational mode; that is, the experimental results of [HEMIm][TFSI]-PVdF mixtures in Figure 1b–d may demonstrate not only the existence of cation–anion and cation–cation clusters, but also increases in the amounts of smaller clusters and isolated cations upon dilution with PVdF.

**Figure 2:**
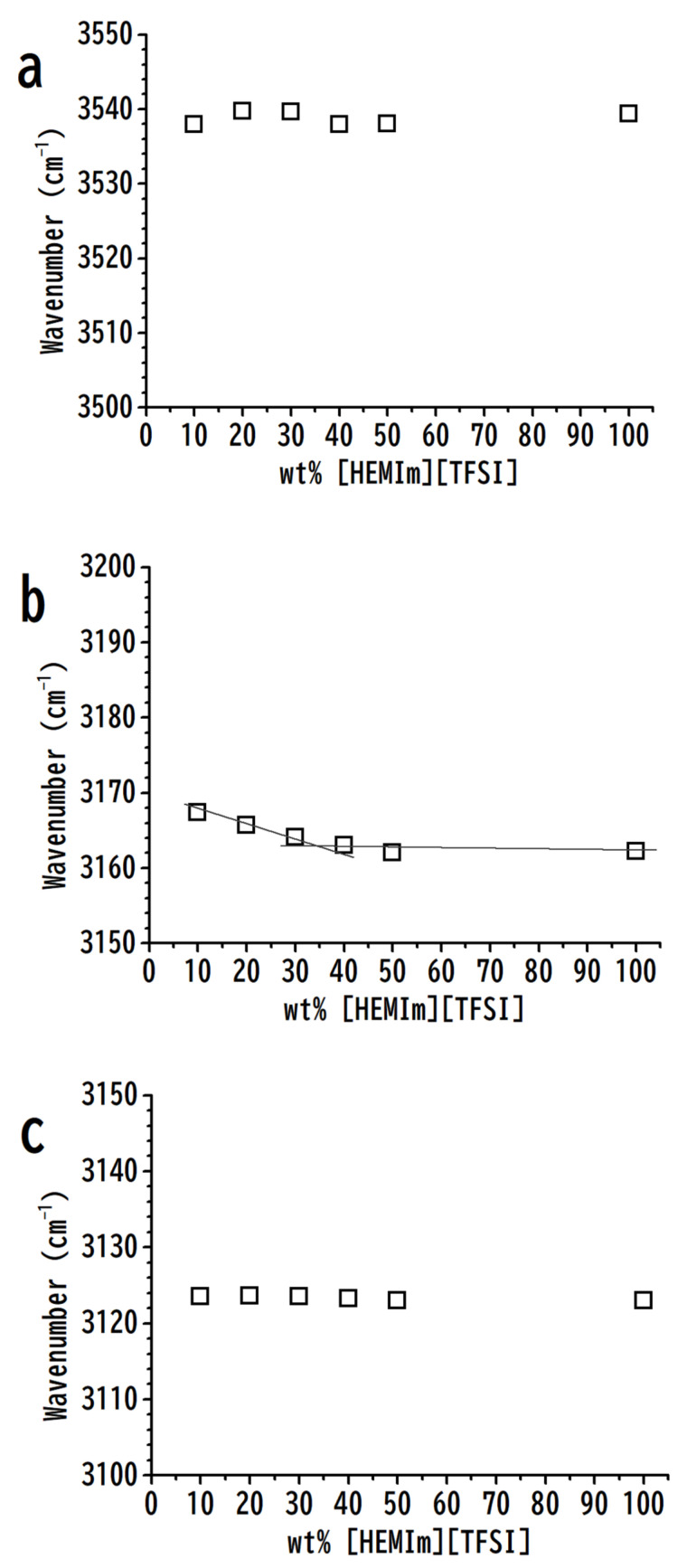
Concentration dependences of (**a**) hydroxy O–H, (**b**) imidazolium C^4,5^–H, and (**c**) imidazolium C^2^–H stretching bands of [HEMIm][TFSI]-PVdF mixtures as a function of the weight percentage of [HEMIm][TFSI].

The concentration dependences of cationic C–H and O–H stretching frequencies of [HEMIm][TFSI]-PVdF mixtures are shown in Figure 2 to illustrate the divisions of [HEMIm][TFSI] associations into smaller clusters by PVdF. In Figure 2a,c, the O–H and C^2^–H stretching band frequencies show negligible changes with various PVdF concentrations. In contrast, the C^4,5^–H peak in Figure 2b show negligible frequency shifts in the region of high [HEMIm][TFSI] concentrations (above 30 wt%) of [HEMIm][TFSI] and non-negligible shifts at diluted [HEMIm][TFSI] concentrations (below 30 wt%). The non-monotonic frequency shifts of the C^4,5^–H vibrations in Figure 2b at various concentrations may reflect the presence of microheterogeneity in the [HEMIm][TFSI] systems. PVdF seems to disturb the intercluster and intracluster associations in the concentrated and diluted regions, respectively. Researchers have concluded that imidazolium C–H absorptions (C^2^–H and C^4,5^–H) reveal different acidic characteristics, while C^2^–H shows stronger hydrogen bonding interactions than C^4,5^–H [39]. In addition, anions prefer to have close contact with the C^2^–H of the imidazolium ring [39]. As PVdF blends into [HEMIm][TFSI], the IL clusters may be divided by the polymer into smaller clusters by the formation of C^4,5^–H⋯PVdF interactions (i.e., breaking the C^4,5^–H⋯anion interaction), leading to the blueshift of C^4,5^–H at low concentrations of [HEMIm][TFSI] for [HEMIm][TFSI]-PVdF mixtures, as shown in Figure 2b.

**Figure 3:**
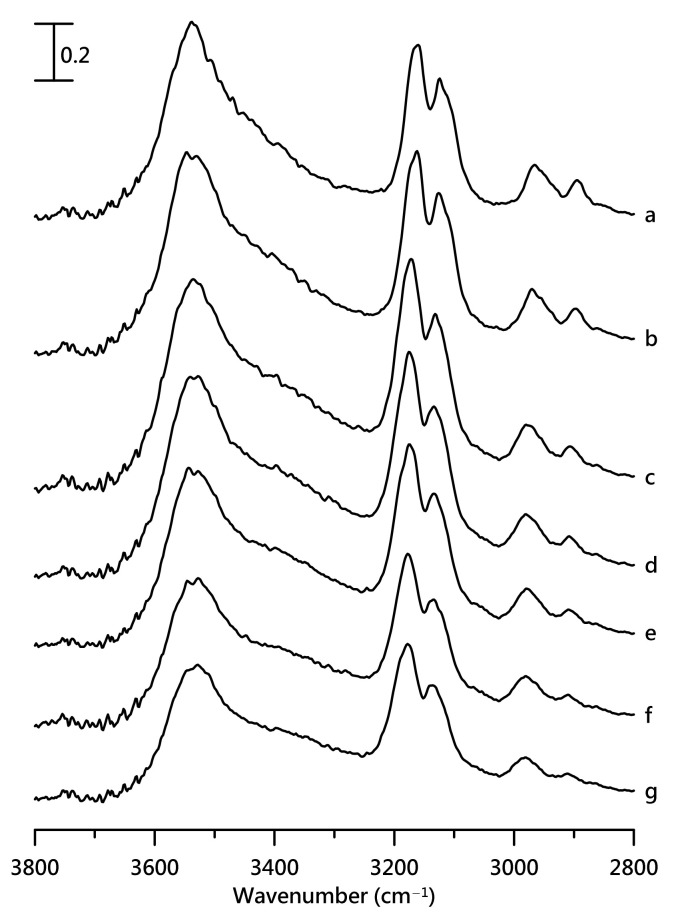
IR spectra of pure [HEMIm][TFSI] at (a) ambient pressure and at (b) 0.4, (c) 0.7, (d) 1.1, (e) 1.5, (f) 1.8, and (g) 2.5 GPa.

Figure 3 shows the IR spectral features of pure [HEMIm][TFSI] at various pressures. As the pressure increases, the C^2^–H and C^4,5^–H bands show a blueshift accompanied by mild-band broadening. In contrast to C–H absorption, the O–H stretching bands show a redshift as the pressure is increased from ambient pressure to 2.5 GPa, as shown in Figure 3. The results in Figure 3 demonstrate that C^2^–H and C^4,5^–H may suffer pressure-induced changes in weak hydrogen-bonding interactions (a blueshift) with anions [32,33,34,35,36]. Conversely, the pressure-induced redshift of the O–H vibrational bands may result from both pressure-enhanced strong hydrogen bonding and the formation of larger cationic clusters (lower frequencies accompanied by band broadening) via pressure-induced association [32,33,34,35,36].

**Figure 4:**
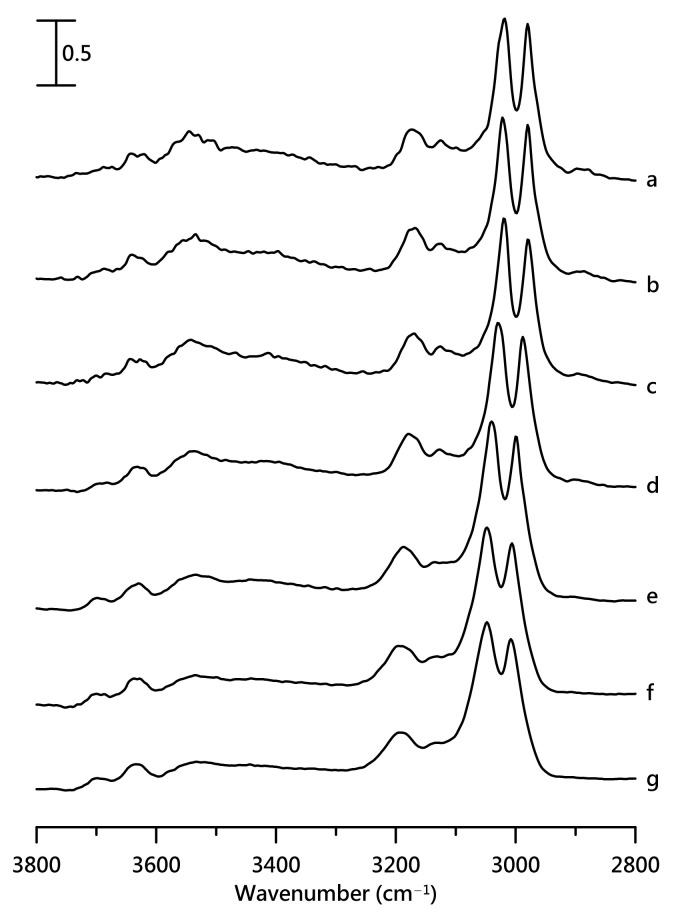
IR spectra of mixture of PVdF containing 10 wt% [HEMIm][TFSI] at (a) ambient pressure and at (b) 0.4, (c) 0.7, (d) 1.1, (e) 1.5, (f) 1.8, and (g) 2.5 GPa.

Figure 4 shows IR spectra of a mixture containing 10 wt% [HEMIm][TFSI] at (Figure 4a) ambient pressure and at (Figure 4b) 0.4, (Figure 4c) 0.7, (Figure 4d) 1.1, (Figure 4e) 1.5, (Figure 4f) 1.8, and (Figure 4g) 2.5 GPa. In Figure 4, the imidazolium C–H absorptions show a blueshift as the pressure increases from ambient pressure in Figure 4a to 2.5 GPa in Figure 4g. The two main O–H stretching bands at ca. 3538 and 3639 cm^−1^ show a redshift as the pressure increases, as shown in Figure 4. The peak broadening of the O–H band at ca. 3538 cm^−1^ becomes obvious at high pressures. We note that the intensity ratio of I_3538_/I_3639_ decreases upon compression. The ratio of I_3538_ and I_3639_ represents the proportions of larger cation–anion clusters and isolated O–H in the [HEMIm][TFSI]-PVdF mixture with 10 wt% [HEMIm][TFSI]. I_3538_/I_3639_ may provide information about the specific local structural changes as high pressure is introduced to the mixture. The decreases in I_3538_/I_3639_ under high pressures suggest that PVdF may further separate the IL clusters into free O–H cations via pressure-enhanced PVdF-IL interactions. A minor band at ca. 3690 cm^−1^ arises at high pressures in Figure 4d–g. The observation of 3640 and 3690 cm^−1^ peaks suggests the presence of at least two local environments for isolated O–H structures due to PVdF-IL interactions under high pressures. Figures S2 and S3 (in the Supplementary Materials) illustrate pressure-dependent IR spectra of the mixture with 20 wt% [HEMIm][TFSI] and pure PVdF, respectively.

**Figure 5:**
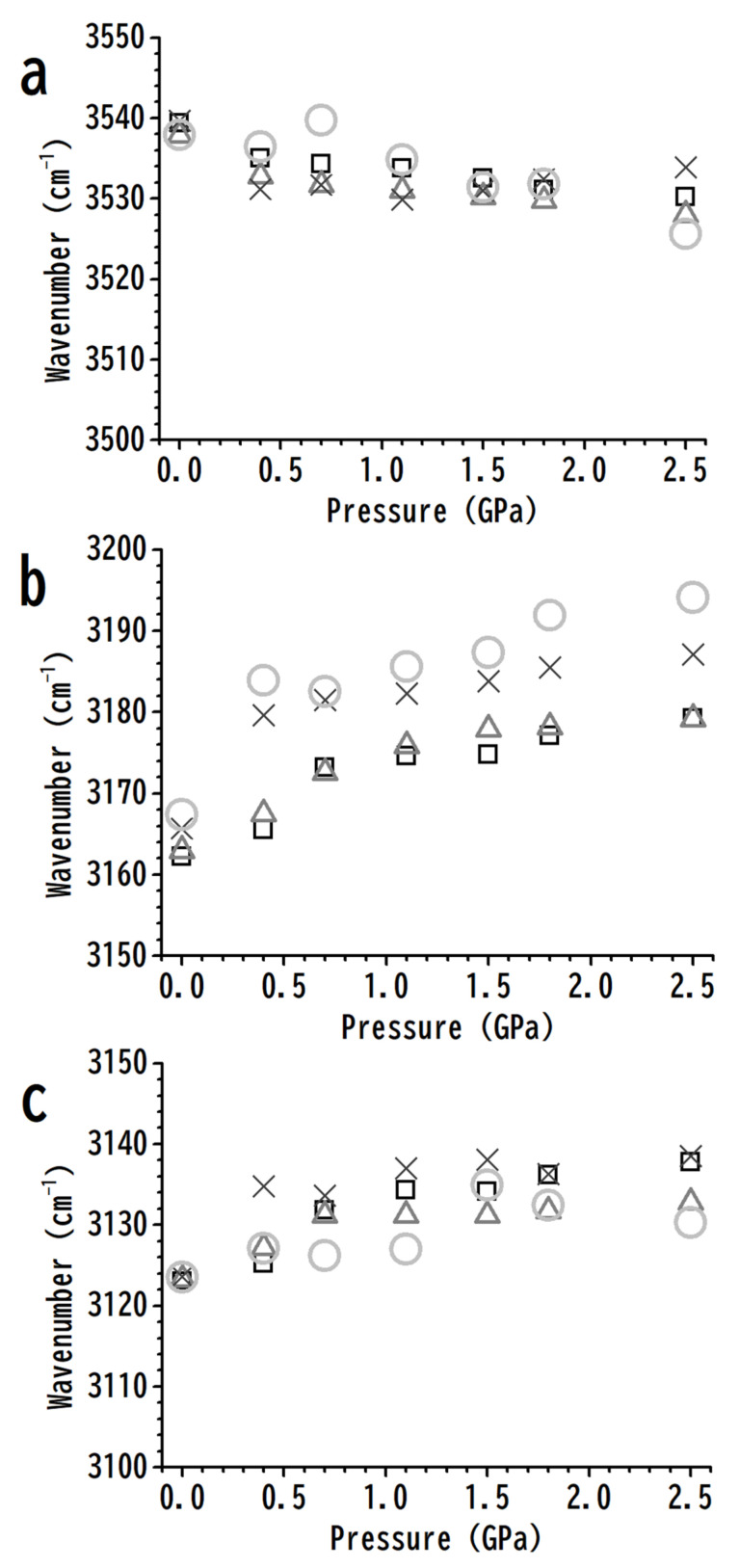
Pressure dependences of the O–H and C–H stretching frequencies of cation O–H (**a**), C^4,5^–H (**b**), and C^2^–H (**c**) for pure [HEMIm][TFSI] (squares) and for [HEMIm][TFSI]-PVdF mixtures containing 40 (triangles), 20 (crosses), and 10 (circles) wt% [HEMIm][TFSI].

The pressure dependences of the O–H, C^4,5^–H, and C^2^–H stretching frequencies for various concentrations are displayed in Figure 5. Figure 5a shows the redshifts of the hydrogen-bonded O–H stretching band upon compression for both pure [HEMIm][TFSI] and mixtures; however, the addition of PVdF does not induce remarkable changes in the O–H stretching frequency in Figure 5a at any pressure. The results in Figure 5a indicate that PVdF may not further influence the local structures of hydrogen-bonded O–H upon compression. The imidazolium ring C^4,5^–H band frequencies increased from 3179 cm^−1^ (pure [HEMIm][TFSI]) to 3194 cm^−1^ (10 wt% mixture) under a pressure of 2.5 GPa. We note that the C^4,5^–H stretching frequencies are 3162 and 3167 cm^−1^ for pure [HEMIm][TSFI] and 10 wt% mixture, respectively, under ambient pressure, as shown in Figure 5b. Traditional hydrogen-bonded O–H⋯anion associations may remain strong under high pressures, as demonstrated in Figure 5a, while the weaker C^4,5^–H⋯anion interactions are likely disturbed by pressure-enhanced IL–PVdF interactions, as shown in Figure 5b. In short, in comparison with pure [HEMIm][TFSI], the addition of polymer material (PVdF) may disturb the local structures of C^4,5^–H in mixtures of [HEMIm][TFSI]-PVdF upon compression, as indicated in Figure 5b. As shown in Figure 5c, the C^2^–H stretching frequencies of pure [HEMIm][TFSI] and [HEMIm][TFSI]-PVdF mixtures demonstrate similar tendencies upon compression; that is, the local environment of C^2^–H may not be easily interfered with by PVdF under high pressures. These observations agree with the fact that C^2^–H has stronger acidity than C^4,5^–H, while C^2^–H⋯anion hydrogen-bonding interactions are more attractive than C^4,5^–H⋯anions.

For pure [HEMIm][TFSI], the imidazolium and anions may involve at least three kinds of associations: O–H⋯anions, C^4,5^–H⋯anions, and C^2^–H⋯anions. As the polymer is added to [HEMIm][TFSI] to form [HEMIm][TFSI]-PVdF mixtures, the weaker interactions between imidazolium and the anion may be disturbed by the PVdF molecule. As shown in Figure 5, the stretching frequencies of C^4,5^–H are significantly influenced by PVdF via pressure-enhanced C^4,5^–H⋯PVdF interactions. The C^4,5^–H⋯anion seems to be the weakest interaction when compared to the O–H⋯anion and C^2^–H⋯anion at high pressures.

**Figure 6:**
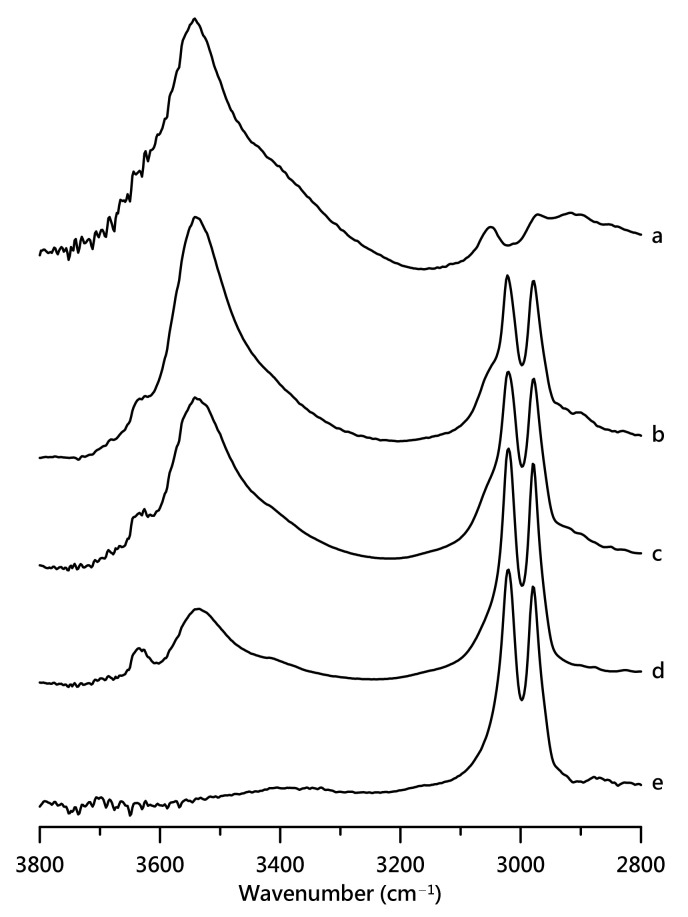
IR spectra of (a) pure choline [TFSI]; mixtures of PVdF containing (b) 30, (c) 20, and (d) 10 wt% choline [TFSI]; and (e) pure PVdF at ambient pressure.

In order to shed more light on the association between cations and anions, choline [TFSI] was studied. Figure 6 shows the IR spectra for pure choline [TFSI], three choline [TFSI]-PVdF mixtures with various concentrations, and pure PVdF obtained at ambient pressure. The spectrum of pure choline [TFSI] in Figure 6a shows broad O–H absorption at ca. 3541 cm^−1^ and alkyl C–H stretching bands located in the region of 800–3100 cm^−1^. The pure PVdF IR spectrum in Figure 6e shows two dominant C–H stretching bands in the region from 2900 to 3150 cm^−1^. Unfortunately, the choline C–H absorption overlaps with the PVdF C–H bands in the region from 2800 to 3100 cm^−1^ (Figure 6); thus, we focused on O–H stretching in the region from 3200 to 3800 cm^−1^ for the mixtures. As choline [TFSI] is diluted by the polymer, an additional peak appears at ca. 3630 cm^−1^ for choline [TFSI]-PVdF mixtures, as is shown in Figure 6b–d. The peak above 3600 cm^−1^ for mixtures of choline [TFSI]-PVdF in Figure 6b–d was regarded as the free O–H stretching of choline cations or partially dissociated clusters for choline [TFSI]. PVdF may tend to change the local structures of the O–H moiety by cutting the hydrogen-bonded network of choline [TFSI] into small clusters or free cations.

**Figure 7:**
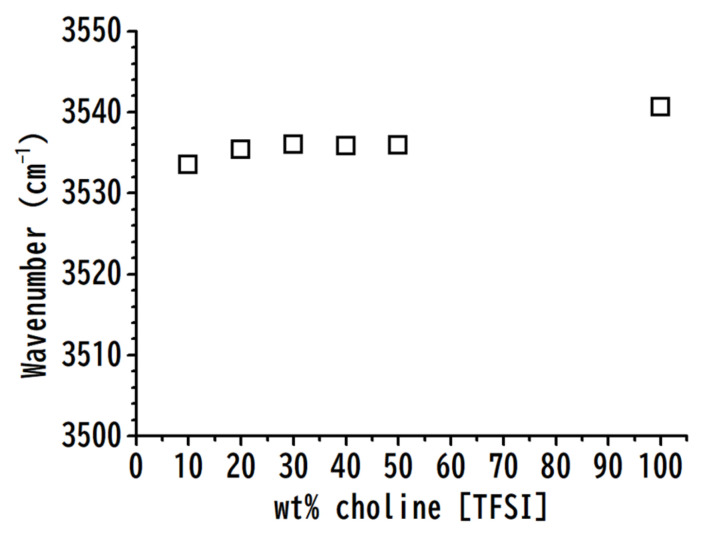
Concentration dependences of hydroxy O–H stretching bands of choline [TFSI]–PVdF mixtures as a function of the weight percentage of choline [TFSI].

Figure 7 illustrates the concentration dependences of the O–H stretching bands of choline [TFSI]-PVdF mixtures as a function of the weight percentage of choline [TFSI]. As shown in Figure 7, the O–H stretching band of pure choline [TFSI] at ca. 3541 cm^−1^ is red-shifted to 3534 cm^−1^ for the mixture with 10 wt% choline [TFSI]. The redshifts of the hydrogen-bonded O–H bands were not observed for the [HEMIm][TFSI]-PVdF mixture (Figure 2a). The [HEMIm] cation has three possible hydrogen-bonding sites for cation–anion interactions (C^4,5^–H⋯anion, C^2^–H⋯anion, and O–H⋯anion), whereas choline [TFSI] has only O–H⋯anions. As the polymer was mixed with choline [TFSI], PVdF may have disturbed the local structure of the hydrogen-bonded O–H of choline, owing to the lack of more favorable options.

**Figure 8:**
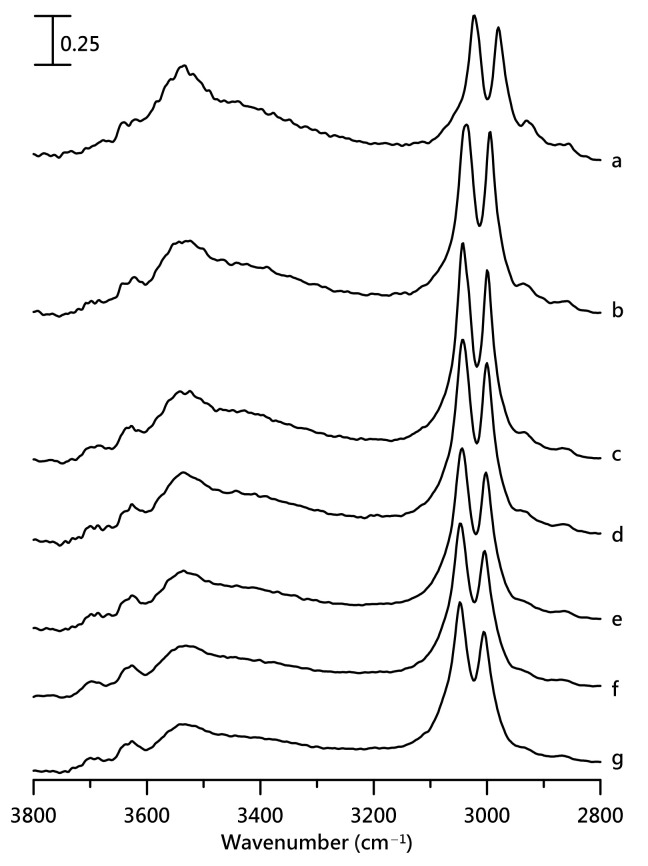
IR spectra of mixture of PVdF containing 10 wt% choline [TFSI] at (a) ambient pressure and at (b) 0.4, (c) 0.7, (d) 1.1, (e) 1.5, (f) 1.8, and (g) 2.5 GPa.

Figure 8 shows the IR spectra of the 10 wt% choline [TFSI]-PVdF mixture under various pressures. At the ambient pressure, the spectrum of the 10 wt% mixture reveals O–H absorptions at 3534 and 3633 cm^−1^, which may be owing to the cation–anion hydrogen bonding network and free-like O–H of choline (Figure 8a). As the pressure increases (Figure 8b–g), the spectra show three distinguishable O–H bands at ca. 3532, 3622, and 3686 cm^−1^, corresponding to hydrogen bonding O–H networks and two free-like O–H structures, respectively. Furthermore, the intensity ratio of hydrogen bonding O–H absorption to free-like O–H absorption (I_3532_/(I_3622_ + I_3686_)) decreases as the pressure increases to 2.5 GPa, as shown in Figure 5g. The decrease in the ratio may indicate that the percentage of free OH increases in the 10 wt% choline [TFSI]-PVdF mixture upon compression. These results may be attributed to the insertion of PVdF into the cation–anion aggregations via pressure-induced interactions, while the polymer molecules may provide at least two surroundings for the free OH group. The IR spectra of pure choline [TFSI] at various pressures are shown in Figure S4.

**Figure 9:**
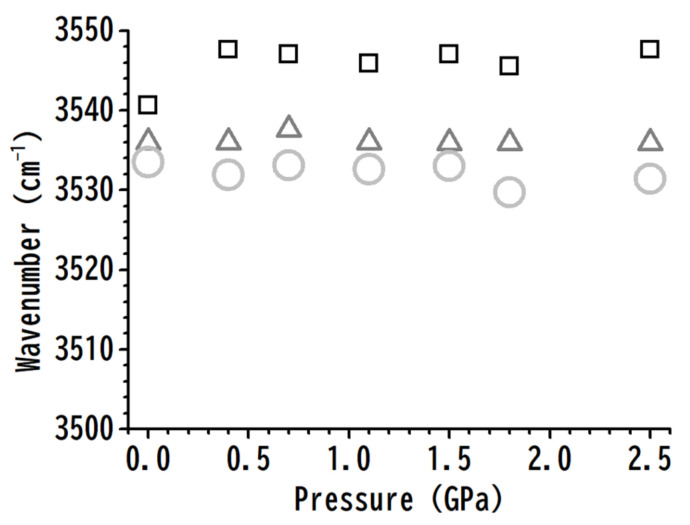
Pressure dependences of the O–H stretching frequencies of cations for pure choline [TFSI] (squares) and choline [TFSI]-PVdF mixtures containing 30 (triangles) and 10 (circles) wt% choline [TFSI].

Figure 9 shows the pressure dependence of the O–H stretching frequencies of pure choline [TFSI] and two choline [TFSI]-PVdF mixtures (containing 30 and 10 wt% choline [TFSI]). In Figure 9, the O–H stretching frequencies reveal mild redshifts from 3541 cm^−1^ (pure choline [TFSI]) to 3534 cm^−1^ (10 wt% mixture) under ambient pressure. In contrast, large redshifts from 3548 cm^−1^ (pure choline [TFSI]) to 3531 cm^−1^ (10 wt% mixture) were detected at a pressure of 2.5 GPa. We note that such significant redshifts under high pressures in Figure 9 are not observed in the O–H of the [HEMIm][TFSI] systems in Figure 5a. In the absence of weaker interactions (such as C^4,5^–H⋯anion), the O–H group is a favorable site for pressure-enhanced cation-PVdF interactions in choline [TFSI]-PVdF mixtures.

## 4. Conclusions

In this study, we demonstrated that the presence of PVdF can influence the clustering structures of [HEMIm][TFSI] and choline [TFSI]. The increase in free –OH absorption at ca. 3634 cm^−1^ for diluted IL-PVdF mixtures suggest an increase in the number of smaller clusters and isolated cations upon dilution at ambient pressure. As the pressure increased, the C^4,5^–H⋯anion associations were likely further disturbed by pressure-enhanced [HEMIm][TFSI]-PVdF interactions. Upon compression, PVdF tended to modify the local structure of the hydrogen bond O–H in choline [TFSI]-PVdF mixtures. The results suggest that high pressure can change the clustering structures and might be successful in modifying the electrochemical properties (such as electric conductivity) of IL-PVdF mixtures. The differences in clustering structures under various pressures may suggest the potential of IL-PVdF mixtures to serve as pressure-dependent electronic switches. Hopefully, the experimental results presented in this study may stimulate further simulation research in the future to rationalize the structural changes of IL-PVdF mixtures in detail under high pressures.

## Data Availability

The data presented in this study are available on request from the corresponding author.

## Supplementary Materials

The following are available online at https://www.mdpi.com/article/10.3390/nano11082099/s1: Figure S1: Structure of [HEMIm][TFSI], choline [TFSI], and PVdF. Figure S2: IR spectra of mixture of PVdF containing 20 wt% [HEMIm][TFSI] at (a) ambient pressure and at (b) 0.4, (c) 0.7, (d) 1.1, (e) 1.5, (f) 1.8, and (g) 2.5 GPa. Figure S3: IR spectra of pure PVdF at (a) ambient pressure and at (b) 0.4, (c) 0.7, (d) 1.1, (e) 1.5, (f) 1.8, and (g) 2.5 GPa. Figure S4: IR spectra of pure choline [TFSI] at (a) ambient pressure and at (b) 0.4, (c) 0.7, (d) 1.1, (e) 1.5, (f) 1.8, and (g) 2.5 GPa.
