# Supplementary material for: Pressure-Dependent Clustering in Ionic-Liquid-Poly (Vinylidene Fluoride) Mixtures: An Infrared Spectroscopic Study
Source: Nanomaterials (Basel). 2021 Aug 18;11(8):2099. doi: 10.3390/nano11082099 (PMC8400791; doi:10.3390/nano11082099)
Supplement: Supplementary file 1 [file nanomaterials-11-02099-s001.zip › nanomaterials-1328428-supplementary.pdf]

Supplementary Materials

Article

Pressure-Dependent Clustering in Ionic-Liquid-Poly (Vinylidene Fluoride) Mixtures: An Infrared Spectroscopic Study

Teng-Hui Wang, Wei-Xiang Wang and Hai-Chou Chang *

Department of Chemistry, National Dong Hwa University, Shoufeng, Hualien 974, Taiwan; 810712101@gms.ndhu.edu.tw (T.-H.W.); 610812112@gms.ndhu.edu.tw (W.-X.W.)

* Correspondence: hcchang@gms.ndhu.edu.tw; Tel.: +886-3-8903585; Fax: +886-3-8900162

Figure S1: Structure of [HEMIm][TFSI], choline [TFSI], and PVdF.

Figure S2: IR spectra of mixture of PVdF containing 20 wt% [HEMIm][TFSI] at (a) ambient pressure and (b) 0.4, (c) 0.7, (d) 1.1, (e) 1.5, (f) 1.8, and (g) 2.5 GPa.

Figure S3: IR spectra of pure PVdF at (a) ambient pressure and (b) 0.4, (c) 0.7, (d) 1.1, (e) 1.5, (f) 1.8, and (g) 2.5 GPa.

Figure S4: IR spectra of pure choline [TFSI] at (a) ambient pressure and (b) 0.4, (c) 0.7, (d) 1.1, (e) 1.5, (f) 1.8, and (g) 2.5 GPa.

1-(2-Hydroxyethyl)-3-methylimidazolium bis(trifluoromethylsulfonyl)imide

[HEMIm][TFSI]

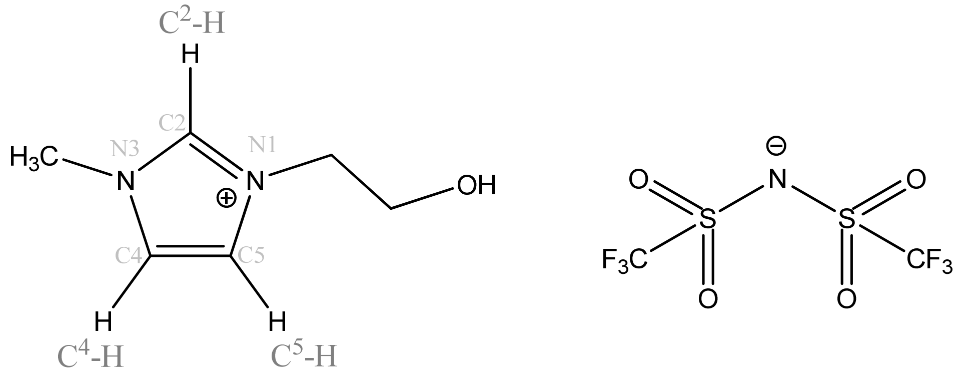

Choline bis(trifluoromethylsulfonyl)imide

Choline [TFSI]

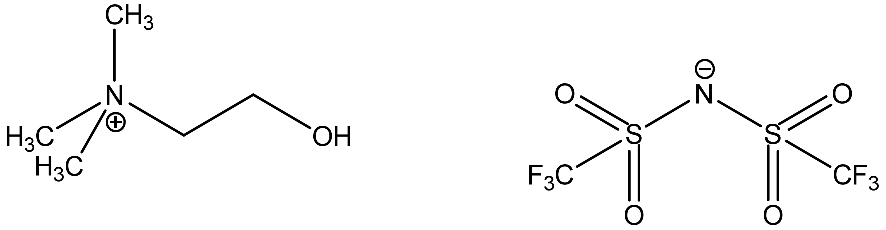

Polyvinylidene fluoride

PVdF

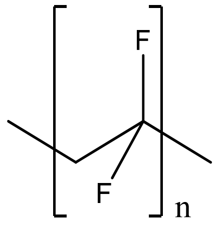

Figure S1. Structure of [HEMIm][TFSI], choline [TFSI], and PVdF

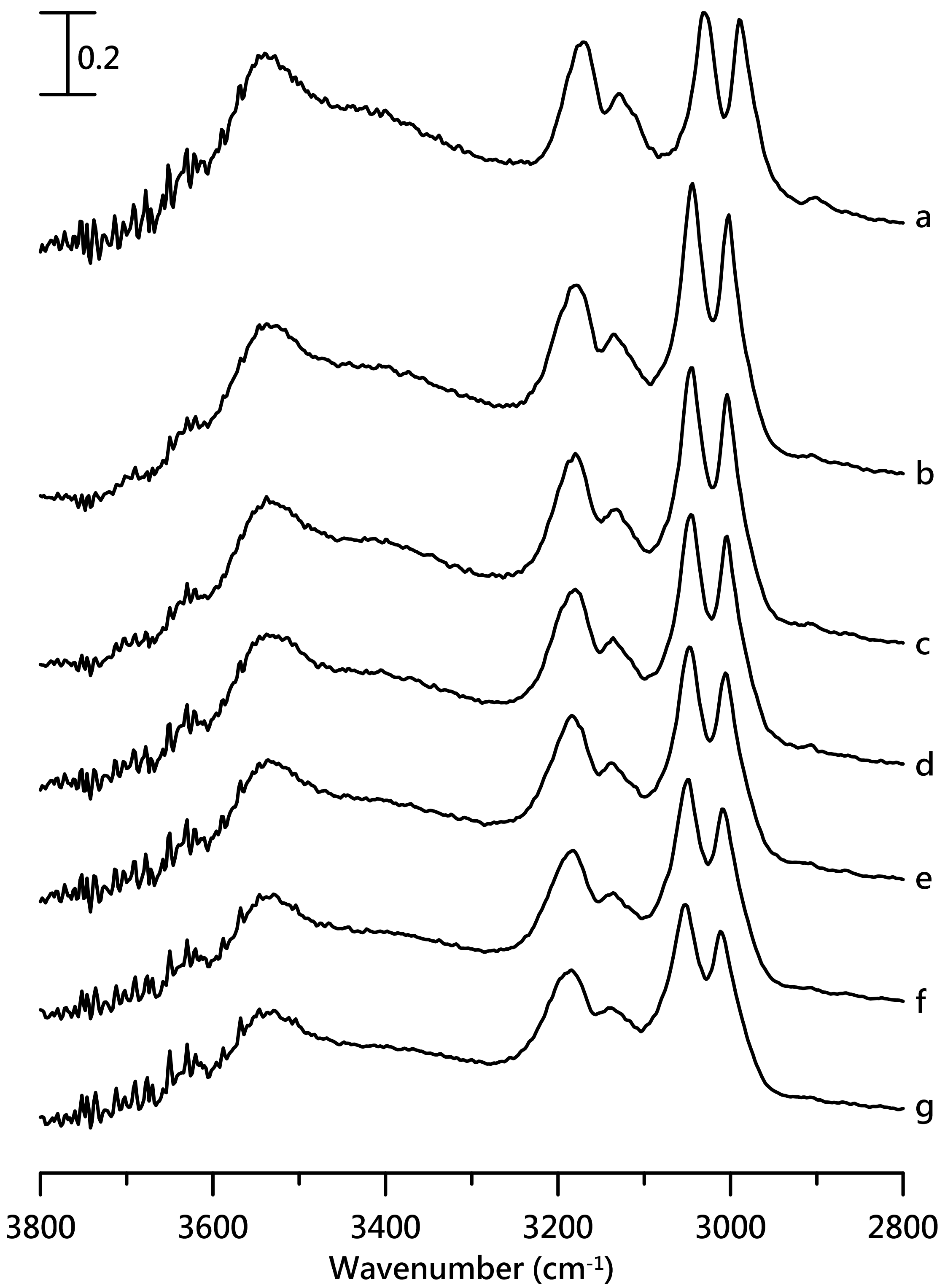

Figure S2. IR spectra of mixture of PVdF containing 20 wt% [HEMIm][TFSI] at (a) ambient pressure and (b) 0.4, (c) 0.7, (d) 1.1, (e) 1.5, (f) 1.8, and (g) 2.5 GPa.

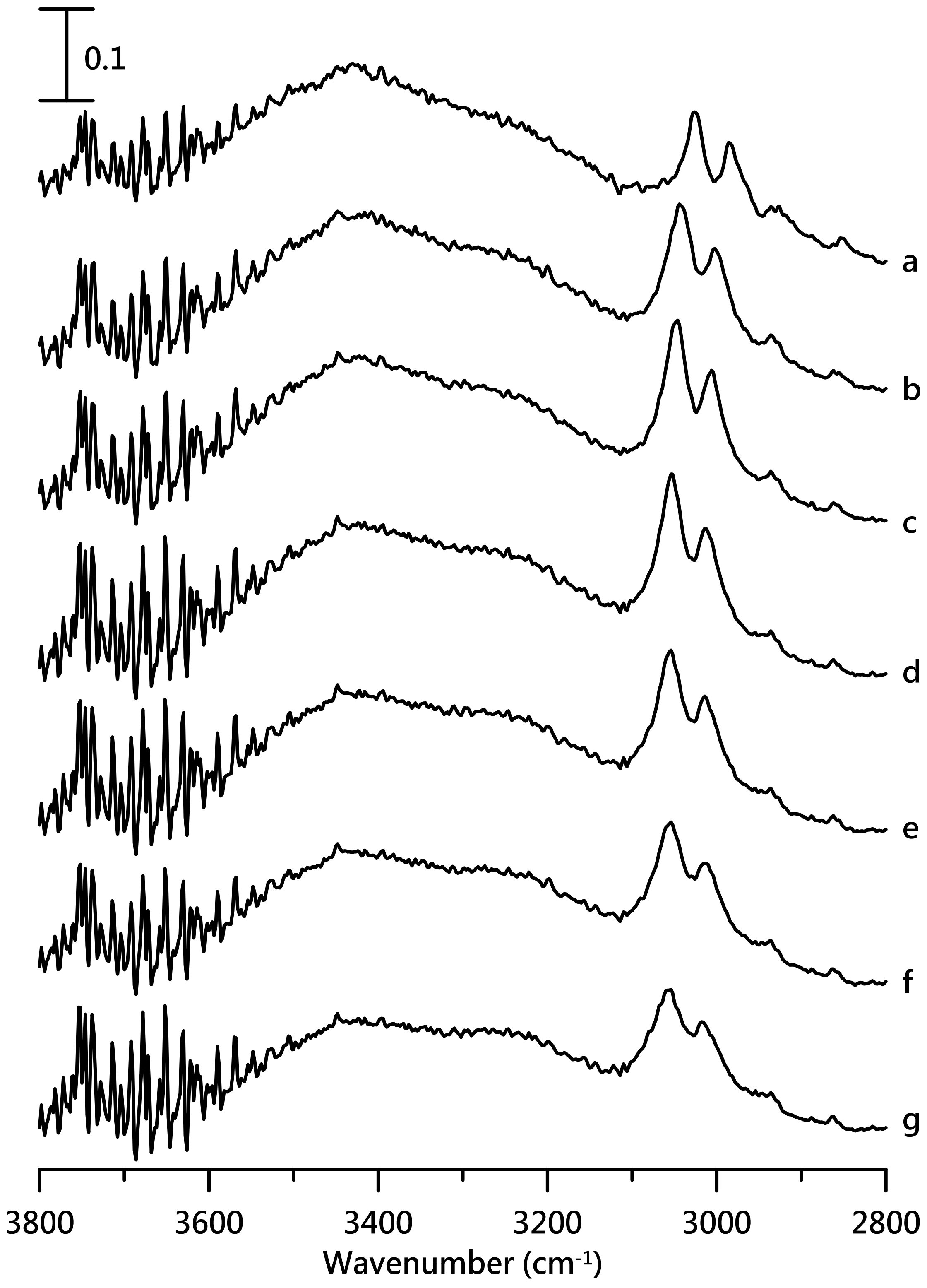

Figure S3. IR spectra of pure PVdF at (a) ambient pressure and (b) 0.4, (c) 0.7, (d) 1.1, (e) 1.5, (f) 1.8, and (g) 2.5 GPa.

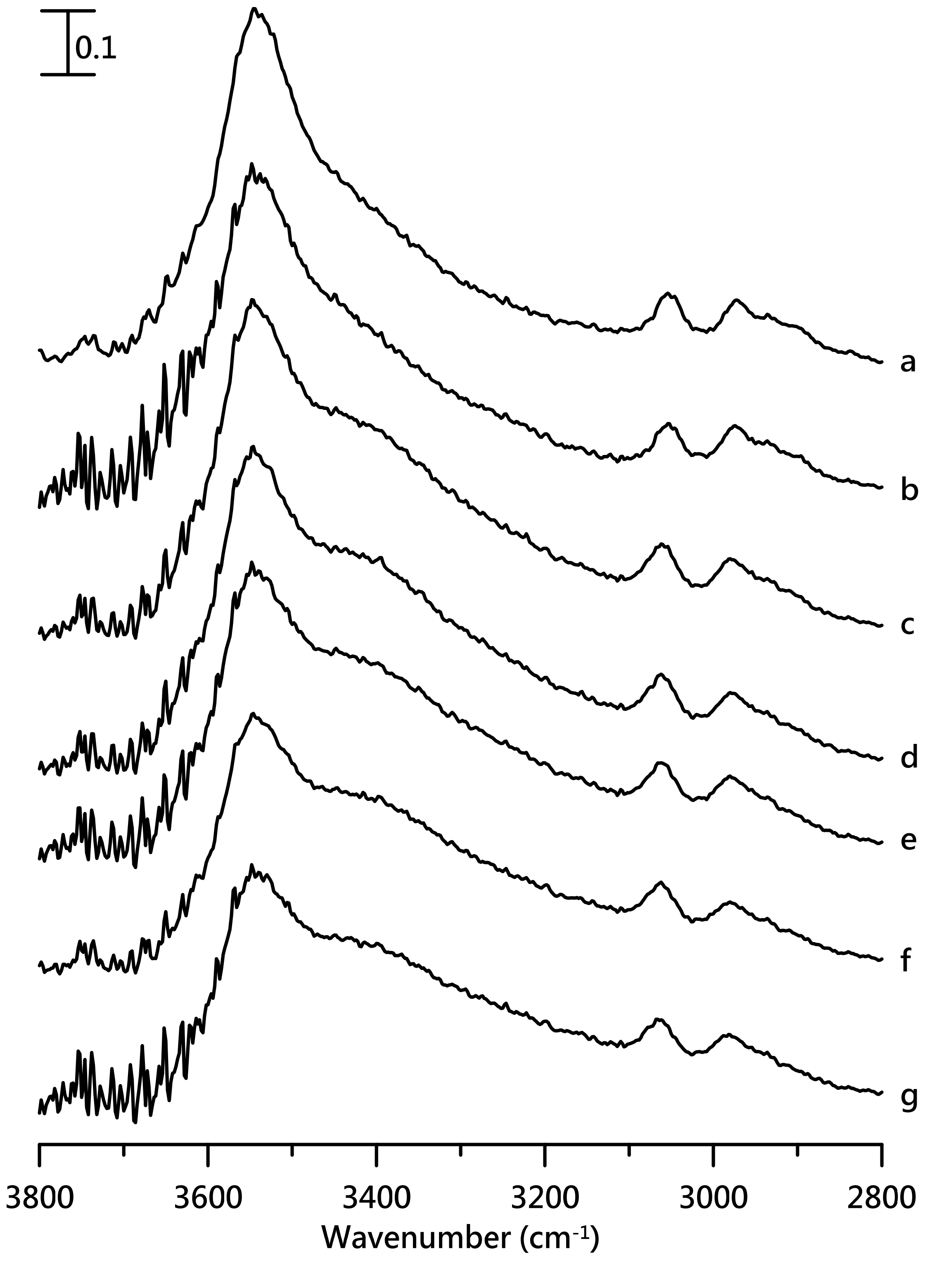

Figure S4. IR spectra of pure choline [TFSI] at (a) ambient pressure and (b) 0.4, (c) 0.7, (d) 1.1, (e) 1.5, (f) 1.8, and (g) 2.5 GPa.
